# Elevated plasma levels of selective cytokines in COVID-19 patients reflect viral load and lung injury

**DOI:** 10.1093/nsr/nwaa037

**Published:** 2020-03-09

**Authors:** Yingxia Liu, Cong Zhang, Fengming Huang, Yang Yang, Fuxiang Wang, Jing Yuan, Zheng Zhang, Yuhao Qin, Xiaoyun Li, Dandan Zhao, Shunwang Li, Shuguang Tan, Zhaoqin Wang, Jinxiu Li, Chenguang Shen, Jianming Li, Ling Peng, Weibo Wu, Mengli Cao, Li Xing, Zhixiang Xu, Li Chen, Congzhao Zhou, William J Liu, Lei Liu, Chengyu Jiang

**Affiliations:** Shenzhen Key Laboratory of Pathogen and Immunity, National Clinical Research Center for Infectious Disease, State Key Discipline of Infectious Disease, Shenzhen Third People's Hospital, Second Hospital Affiliated to Southern University of Science and Technology, Shenzhen 518112, China; Hefei National Laboratory for Physical Sciences at the Microscale and School of Life Sciences, University of Science and Technology of China, Hefei 230027, China; The State Key Laboratory of Medical Molecular Biology, Institute of Basic Medical Sciences, Chinese Academy of Medical Sciences, Department of Biochemistry, Peking Union Medical College, Beijing 100005, China; The State Key Laboratory of Medical Molecular Biology, Institute of Basic Medical Sciences, Chinese Academy of Medical Sciences, Department of Biochemistry, Peking Union Medical College, Beijing 100005, China; Shenzhen Key Laboratory of Pathogen and Immunity, National Clinical Research Center for Infectious Disease, State Key Discipline of Infectious Disease, Shenzhen Third People's Hospital, Second Hospital Affiliated to Southern University of Science and Technology, Shenzhen 518112, China; Shenzhen Key Laboratory of Pathogen and Immunity, National Clinical Research Center for Infectious Disease, State Key Discipline of Infectious Disease, Shenzhen Third People's Hospital, Second Hospital Affiliated to Southern University of Science and Technology, Shenzhen 518112, China; Shenzhen Key Laboratory of Pathogen and Immunity, National Clinical Research Center for Infectious Disease, State Key Discipline of Infectious Disease, Shenzhen Third People's Hospital, Second Hospital Affiliated to Southern University of Science and Technology, Shenzhen 518112, China; Shenzhen Key Laboratory of Pathogen and Immunity, National Clinical Research Center for Infectious Disease, State Key Discipline of Infectious Disease, Shenzhen Third People's Hospital, Second Hospital Affiliated to Southern University of Science and Technology, Shenzhen 518112, China; The State Key Laboratory of Medical Molecular Biology, Institute of Basic Medical Sciences, Chinese Academy of Medical Sciences, Department of Biochemistry, Peking Union Medical College, Beijing 100005, China; The State Key Laboratory of Medical Molecular Biology, Institute of Basic Medical Sciences, Chinese Academy of Medical Sciences, Department of Biochemistry, Peking Union Medical College, Beijing 100005, China; The State Key Laboratory of Medical Molecular Biology, Institute of Basic Medical Sciences, Chinese Academy of Medical Sciences, Department of Biochemistry, Peking Union Medical College, Beijing 100005, China; The State Key Laboratory of Medical Molecular Biology, Institute of Basic Medical Sciences, Chinese Academy of Medical Sciences, Department of Biochemistry, Peking Union Medical College, Beijing 100005, China; The NHC Key Laboratory of Biosafety, National Institute for Viral Disease Control and Prevention, China CDC, Beijing 102206, China; Shenzhen Key Laboratory of Pathogen and Immunity, National Clinical Research Center for Infectious Disease, State Key Discipline of Infectious Disease, Shenzhen Third People's Hospital, Second Hospital Affiliated to Southern University of Science and Technology, Shenzhen 518112, China; Shenzhen Key Laboratory of Pathogen and Immunity, National Clinical Research Center for Infectious Disease, State Key Discipline of Infectious Disease, Shenzhen Third People's Hospital, Second Hospital Affiliated to Southern University of Science and Technology, Shenzhen 518112, China; Shenzhen Key Laboratory of Pathogen and Immunity, National Clinical Research Center for Infectious Disease, State Key Discipline of Infectious Disease, Shenzhen Third People's Hospital, Second Hospital Affiliated to Southern University of Science and Technology, Shenzhen 518112, China; Shenzhen Key Laboratory of Pathogen and Immunity, National Clinical Research Center for Infectious Disease, State Key Discipline of Infectious Disease, Shenzhen Third People's Hospital, Second Hospital Affiliated to Southern University of Science and Technology, Shenzhen 518112, China; Shenzhen Key Laboratory of Pathogen and Immunity, National Clinical Research Center for Infectious Disease, State Key Discipline of Infectious Disease, Shenzhen Third People's Hospital, Second Hospital Affiliated to Southern University of Science and Technology, Shenzhen 518112, China; Shenzhen Key Laboratory of Pathogen and Immunity, National Clinical Research Center for Infectious Disease, State Key Discipline of Infectious Disease, Shenzhen Third People's Hospital, Second Hospital Affiliated to Southern University of Science and Technology, Shenzhen 518112, China; Shenzhen Key Laboratory of Pathogen and Immunity, National Clinical Research Center for Infectious Disease, State Key Discipline of Infectious Disease, Shenzhen Third People's Hospital, Second Hospital Affiliated to Southern University of Science and Technology, Shenzhen 518112, China; Shenzhen Key Laboratory of Pathogen and Immunity, National Clinical Research Center for Infectious Disease, State Key Discipline of Infectious Disease, Shenzhen Third People's Hospital, Second Hospital Affiliated to Southern University of Science and Technology, Shenzhen 518112, China; Shenzhen Key Laboratory of Pathogen and Immunity, National Clinical Research Center for Infectious Disease, State Key Discipline of Infectious Disease, Shenzhen Third People's Hospital, Second Hospital Affiliated to Southern University of Science and Technology, Shenzhen 518112, China; Shenzhen Key Laboratory of Pathogen and Immunity, National Clinical Research Center for Infectious Disease, State Key Discipline of Infectious Disease, Shenzhen Third People's Hospital, Second Hospital Affiliated to Southern University of Science and Technology, Shenzhen 518112, China; Hefei National Laboratory for Physical Sciences at the Microscale and School of Life Sciences, University of Science and Technology of China, Hefei 230027, China; The NHC Key Laboratory of Biosafety, National Institute for Viral Disease Control and Prevention, China CDC, Beijing 102206, China; Shenzhen Key Laboratory of Pathogen and Immunity, National Clinical Research Center for Infectious Disease, State Key Discipline of Infectious Disease, Shenzhen Third People's Hospital, Second Hospital Affiliated to Southern University of Science and Technology, Shenzhen 518112, China; The State Key Laboratory of Medical Molecular Biology, Institute of Basic Medical Sciences, Chinese Academy of Medical Sciences, Department of Biochemistry, Peking Union Medical College, Beijing 100005, China

**Keywords:** 2019-nCoV, COVID-19, cytokine storm, IP-10, IL-17

## Abstract

A recent outbreak of pneumonia in Wuhan, China was found to be caused by a 2019 novel coronavirus (2019-nCoV or SARS-CoV-2 or HCoV-19). We previously reported the clinical features of 12 patients with 2019-nCoV infections in Shenzhen, China. To further understand the pathogenesis of COVID-19 and find better ways to monitor and treat the disease caused by 2019-nCoV, we measured the levels of 48 cytokines in the blood plasma of those 12 COVID-19 patients. Thirty-eight out of the 48 measured cytokines in the plasma of 2019-nCoV-infected patients were significantly elevated compared to healthy individuals. Seventeen cytokines were linked to 2019-nCoV loads. Fifteen cytokines, namely M-CSF, IL-10, IFN-α2, IL-17, IL-4, IP-10, IL-7, IL-1ra, G-CSF, IL-12, IFN-γ, IL-1α, IL-2, HGF and PDGF-BB, were strongly associated with the lung-injury Murray score and could be used to predict the disease severity of 2019-nCoV infections by calculating the area under the curve of the receiver-operating characteristics. Our results suggest that 2019-nCoV infections trigger extensive changes in a wide array of cytokines, some of which could be potential biomarkers of disease severity of 2019-nCoV infections. These findings will likely improve our understanding of the immunopathologic mechanisms of this emerging disease. Our results also suggest that modulators of cytokine responses may play a therapeutic role in combating the disease once the functions of these elevated cytokines have been characterized.

## INTRODUCTION

Since the outbreak of the 2019 novel coronavirus (2019-nCoV) infections, also named ‘severe acute respiratory syndrome coronavirus 2’ (SARS-CoV-2) or ‘2019 human coronavirus’ (HCoV-19) [1,2] in December 2019 in Wuhan, Hubei Province, China [3–10], the number of infected persons has exceeded 70 000 and already surpassed that of the previous severe acute respiratory syndrome (SARS) epidemic [11]. In this study, we often use the older nomenclature, 2019-nCoV, to avoid confusion when making comparisons between COVID-19 and SARS. Hitherto, reflecting the geographical reach of the pandemic-prone coronaviruses, 2019-nCoV-infected cases have been reported in the Western Pacific, Southeast Asia, Europe, the Americas, eastern Mediterranean regions and other parts of the world [12–16]. The mortality rate is 15% for a case series of 41 hospitalized patients with 2019-nCoV infections in Wuhan, China [5], compared with 10% for SARS-CoV and 37% for Middle East respiratory syndrome coronavirus (MERS-CoV) [17]. Although 2019-nCoV, the seventh reported human-infecting member of the family coronaviridae, which also includes SARS-CoV [18] and MERS-CoV [19,20], has been identified as the causative agent, the immunopathologic mechanisms of 2019-nCoV-associated diseases have not been elucidated.

One prominent feature of 2019-nCoV infections is the presence of acute respiratory distress syndrome (ARDS) in a sizable proportion of the documented 2019-nCoV-infected cases: 29% (12/41) in the series by Huang *et al.* [5], 17% in a series of 99 cases of 2019-nCoV-induced pneumonia (COVID-19) [21] and 50% (6/12) in the series of this study [22]. An elevated production of proinflammatory cytokines/chemokines [23], or even hypercytokinemia [24,25], which is also known as a cytokine storm, was present in SARS-CoV and MERS-CoV infections [18,19,26] and contributes to acute lung injury and development of ARDS [23]. In this study, we examined the plasma cytokine/chemokine profiles of 12 patients with laboratory-confirmed 2019-nCoV infections in Shenzhen China [22] versus 8 healthy subjects, 8 severe bacterial pneumonia patients and 8 patients with influenza virus A H7N9 infections (Supplementary Table 1).

## RESULTS

We used the Bio-Plex Pro Human Cytokine Screening Panel to measure levels of 48 key cytokines/chemokines in blood plasma in the 12 COVID-19 patients who were classified as mild (M) and severe (S) subtypes, in comparison with healthy subjects (Supplementary Table 2). Our results revealed extensive and significant increases in 38 cytokines/chemokines in blood plasma in COVID-19-S patients versus healthy individuals, suggesting the occurrence of hypercytokinemia in COVID-19-S pneumonia patients (Supplementary Table 2). Overall, the plasma cytokine/chemokine levels in COVID-19-S patients were lower than in H7N9 influenza virus A-infected patients, but moderately higher than in patients with severe bacterial pneumonia (Supplementary Table 2). In addition, the plasma cytokine/chemokine levels were higher in Week 2 than in Week 1 from symptomatic onset of 2019-nCoV infections (Supplementary Table 3). The plasma cytokine/chemokine levels were comparable between Days 8 and 14 since illness onset and Day 15 after illness onset, indicating persistent elevations of plasma cytokines/chemokines at the later stage of the disease (Supplementary Table 3), which are consistent with the clinical features of 2019-nCoV infections [3,4,22].

**Figure 1.**
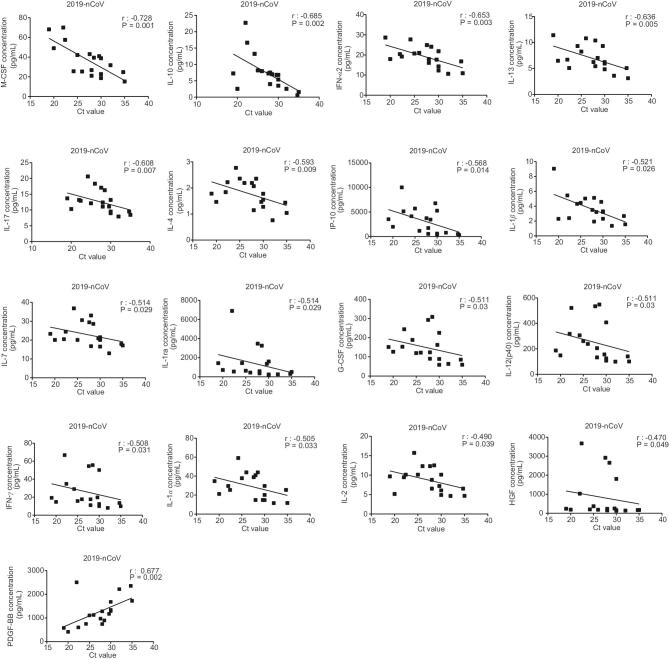
Correlation between cytokine plasma levels and viral Ct value in patients with 2019-nCoV infections. The plasma levels of cytokines (M-CSF, IL-10, IFN-α2, IL-13, IL-17, IL-4, IP-10, IL-1β, IL-7, IL-1ra, G-CSF, IL-12 (p40), IFN-γ, IL-1α, IL-2, HGF and PDGF-BB) were measured in a total of 25 blood samples from 12 patients with 2019-nCoV infections. The viral titers were detected in 18 samples from 10 patients infected with 2019-nCoV. Spearman's rank correlation analysis (r) was used for linear-correlation analysis.

We quantified viral RNA loads in throat swabs, sputum and bronchoalveolar lavage fluid (BALF) samples by quantitative reverse transcription polymerase chain reaction (qRT-PCR) (Supplementary Table 4). Our Spearman’s correlation analysis revealed that 2019-nCoV viral load was highly positively associated with the plasma levels of 16 cytokines (M-CSF, IL-10, IFN-α2, IL-13, IL-17, IL-4, IP-10, IL-1β, IL-7, IL-1ra, G-CSF, IL-12, IFN-γ, IL-1α, IL-2 and HGF) and negatively associated with PDGF-BB (Fig 1). These findings suggest that 2019-nCoV infection is associated with an elevated production of a wide array of cytokines/chemokines in the plasma of 2019-nCoV-infected patients.

**Figure 2.**
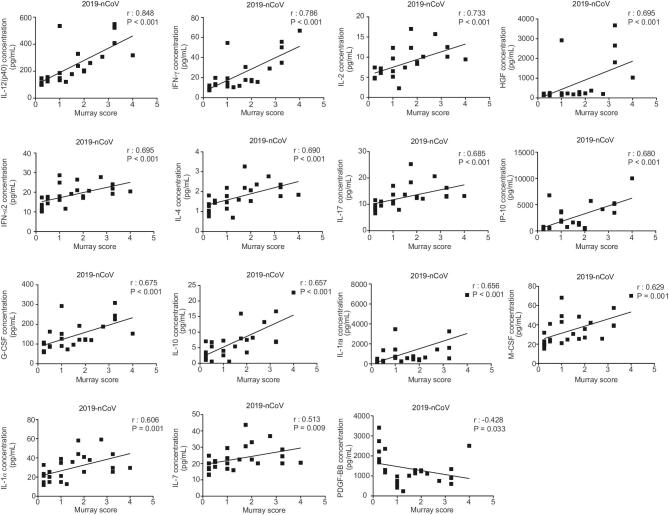
Murray score highly correlates with plasma cytokine levels in patients with 2019-nCoV infections. The plasma levels of cytokines (IL-12, IFN-γ, IL-2, HGF, IFN-α2, IL-4, IL-17, IP-10, G-CSF, IL-10, IL-1ra, M-CSF, IL-1α, IL-7 and PDGF-BB) were measured in a total of 25 blood samples from 12 patients with 2019-nCoV infections. Clinical indicators from the same day as the blood-sample collection were used to calculate the Murray score (an indicator of lung-injury severity). Spearman's rank correlation analysis (r) was used for linear-correlation analysis.

**Figure 3.**
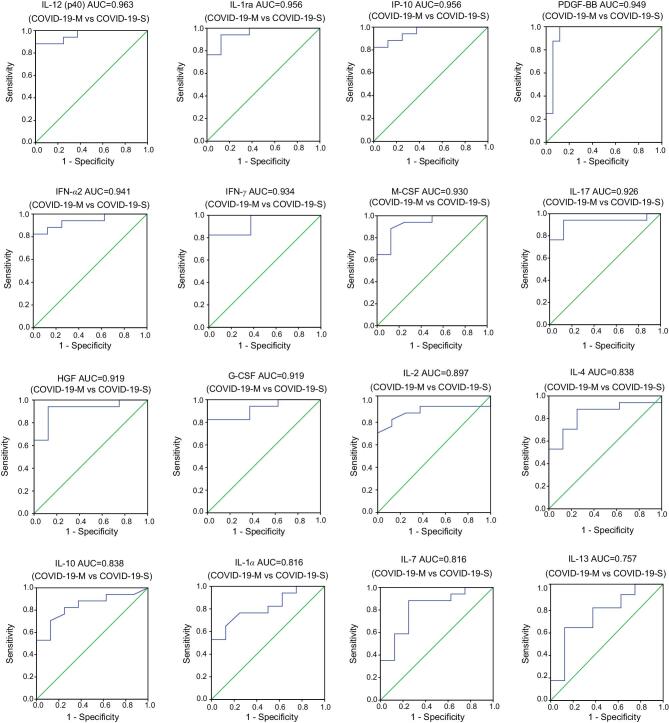
The ROC curve of plasma cytokine levels for patients with mild and severe 2019-nCoV-infections. The area under the receiver-operating characteristic (ROC) curve (AUC) of the plasma cytokine levels (IL-12, IL-1ra, IP-10, PDGF-BB, IFN-α2, IFN-γ, M-CSF, IL-17, HGF, G-CSF, IL-2, IL-4, IL-10, IL-1α and IL-7) was estimated in 8 samples from 4 mild COVID-19 (COVID-19-M) patients and 17 samples from 8 severe COVID-19 (COVID-19-S) patients. The *P*-values of all AUC for plasma cytokine levels were <0.05.

**Figure 4.**
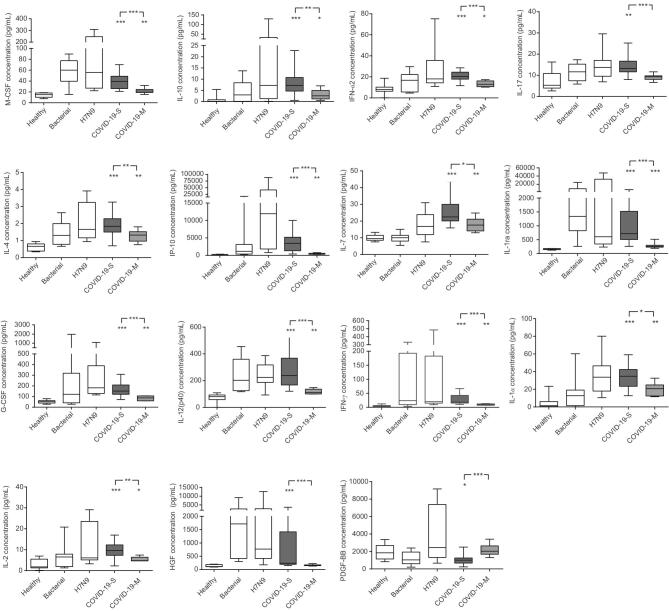
Plasma cytokine levels in healthy controls, severe bacteria pneumonia patients, H7N9-infected patients and 2019-nCoV-infected patients. The plasma levels of cytokines (M-CSF, IL-10, IFN-α2, IL-17, IL-4, IP-10, IL-7, IL-1ra, G-CSF, IL-12 (p40), IFN-γ, IL-1α, IL-2, HGF and PDGF-BB) were measured in 8 samples from 8 healthy controls, 8 samples from 8 severe bacteria pneumonia patients, 8 samples from 8 H7N9-infected patients, 8 samples from 4 2019-nCoV-infected patients with mild illness (COVID-19-M) and 17 samples from 8 patients with severe illness (COVID-19-S). Detailed information is shown in Supplementary Table 2. *P < 0.05; ^**^P < 0.01; ^***^P < 0.001.

Using Spearman’s rank coefficient correlation analysis, we discovered a strong positive linear association between the plasma levels of 14 cytokines (IL-12, IFN-γ, IL-2, HGF, IFN-α2, IL-4, IL-17, IP-10, G-CSF, IL-10, IL-1ra, M-CSF, IL-1α and IL-7) in 2019-nCoV-infected patients and lung-injury Murray score, and a negative association between PDGF-BB and Murray score (Fig. 2). The area under the curve (AUC) of the receiver-operating characteristic (ROC) was >0.8 for each of these 15 cytokines, indicating that these cytokines could predict the disease severity of 2019-nCoV infections (Fig. 3). Furthermore, the plasma cytokines/chemokines levels in COVID-19-S patients were significantly different from those in mild COVID-19 (COVID-19-M) pneumonia patients (Fig. 4). Our data suggest that these 15 cytokines may be biomarkers for disease severity in 2019-nCoV-infected patients.

## DISCUSSION

In summary, we have demonstrated that a cytokine storm likely occurred in 2019-nCoV-infected patients. There were significant elevations in 38 out of 48 plasma cytokines/chemokines in patients with severe COVID-19 pneumonia versus healthy individuals. We discovered that 15 cytokines are linearly associated with lung injury (Murray score) and may be potential biomarkers for disease severity. These cytokines include IFN-α2, IFN-γ, IL1ra, IL2, 4, 7, 10, 12 and 17, chemokine IP-10, as well as G-CSF and M-CSF. It is interesting that the levels of proinflammatory cytokines Th1, Th2 and Th17 were all increased.

A previous study reported the levels of 27 cytokines/chemokine in the blood samples of COVID-19 patients [5]. The profile of these 27 cytokines and chemokines is similar to that in this study. Furthermore, we reported here an additional 21 cytokines/chemokines, including IL-12(p40), IL-18, IFN-α2, MIG, TNF-β, IL-1α, IL-2Rα, IL-3, IL-16, CTACK, GRO-α, HGF, LIF, MCP-3, MIF, b-NGF, M-CSF, SCF, SCGF-β, SDF-1α and TRAIL. We identified 15 increased cytokines/chemokines linearly associated with viral load and lung injury.

A previous study (2005) demonstrated elevation of IP-10, IL-6, IL-8, MCP1 and MIP-1α in serum from SARS-CoV-infected patients [18]. Later studies (2018) showed a significant increase in IL-10, 15 and 17 as well as IFN α2 and γ in plasma samples collected from MERS-CoV-infected patients [19, 20]. Assuming that the levels of soluble cytokines in serum are identical to those in plasma

(less processed blood samples), the increased level of cytokines in blood samples from SARS-CoV and MERS-CoV-infected patients [18,19,26–29] appeared higher than the cytokine levels in the plasma of 2019-nCoV-infected patients. Most of the 48 plasma cytokines/chemokines tested in this study were not reported in plasma samples of SARS-CoV- and MERS-CoV-infected patients. However, levels of TNF-α, IL-12 (p70), IL-13 and IL-15 in plasma from 2019-nCoV-infected patients seemed markedly higher than those from SARS-CoV- and MERS-CoV-infected patients, while the levels of other critical cytokines/chemokines including IP-10 and MIG were approximately in the same range [18, 19, 26–29]. The most extreme case of hypercytokinemia detected in humans may come from blood samples of patients infected with avian influenza A virus. In some cases, hypercytokinemia factors have been associated with IP-10, MCP-1, MIG and IL-8 of patients infected with H5N1 virus [30], as well as MIF, SCF, MCP-1, HGF, SCGF-beta, IP-10, IL-18 and IFN-γ in patients infected with H7N9 virus [31]. Comparing the levels of 48 cytokines/chemokines tested in blood samples from COVID-19-S patients to those from H7N9 infected patients, the difference in the levels of most of the cytokines/chemokines did not reach statistical significance (Supplementary Table 2). Taken together,

our data indicate an exaggerated cytokine presence in plasma from COVID-19-S patients.

These elevated cytokines/chemokines in hosts have different functions in response to pathogen infections. Antiviral cytokines, such as interferons, are ‘good’ in that raised levels enhance host immunity and help eliminate the pathogens. Proinflammatory cytokines, such as IL-6 secreted by monocytes, are ‘bad’ and trigger signaling cascades of multiple cytokines leading to tissue damage and organ failure [32,33]. A cytokine storm including elevated IP-10, IL-17A and basic fibroblast growth factor (FGF2) was reported in serum samples from Swine Origin influenza A virus H1N1 (S-OIV)-infected patients [34–36]. Using genetic-deficient mice infected with S-OIV, our previous studies showed that mice lung pathology was alleviated in IP-10- and IL-17A-deficient mice and exacerbated in FGF2-deficient mice. After elucidating the molecular mechanisms of elevated cytokines/chemokines IP-10, IL-17A and FGF2 in the infected mice models, respectively, we demonstrated that monoclonal antibodies against IP-10 and IL-17A, as well as recombinant FGF2,

could alleviate acute lung injury in mice induced by S-OIV infections [34–36].

Although the mechanisms of cytokine-mediated communications in COVID-19 patients are largely unknown, attempts to use cytokines or cytokine inhibitors therapeutically have been increasingly successful [37,38]. The anti-pathogenic cytokines, such as interferon α and γ, have been frequently used clinically despite their unpleasant side effects [39–41] and interferon α is an officially recommended drug for the treatment of COVID-19 patients in the China National Health Commission Guidelines for Diagnosis and Treatment of COVID-19 Pneumonia [42]. Blocking pro-pathogenic cytokines has been used clinically and in clinical trials for the treatment of autoimmune or autoinflammatory diseases (including monoclonal antibodies

against IL-1 [43], IL-10 [37], IL-12 [44], IL-17 [36] and IP-10 [35]). Existing pharmaceutic modulators of these cytokines might be repurposed to attenuate hypercytokinemia in COVID-19 patients.

Biopsy samples of lung, liver and heart tissue taken from an autopsy of a COVIP-19 patient were analysed [45]. The pathological features of COVID-19 were similar to those seen in SARS-CoV- and MERS-CoV-infected patients [45]. In addition, flow cytometric analysis of a blood sample from this COVID-19 patient revealed that CD4 and CD8 T-cells were hyperactivated and proinflammatory CCR4+ CCR6+ Th17 in CD4 T-cells markedly increased [45]. These data indicate that the severe immune injury in this patient may, in part, be the consequence of the overactivated T-cells and other immune cells.

The excessive host immune responses mediated by pathogenic T-cells and inflammatory CD14+ CD16+ monocytes that express high levels of IL-6 in blood samples of COVID-19-S patients were also observed in a recent study [46]. This study did not measure the levels of IL-6 in blood plasma or serum directly, but is consistent by our observation of elevated levels of IL-6 in COVID-19-S patients. A moderate but significant elevation of IL-6 in the plasma of COVID-19-S patients might be an indicator of hypercytokinemia in lung and other organs, and intervention of the IL-6-mediated pathological cascade may reduce or alleviate the immune attack found in the lungs of COVID-19-S patients. Therefore, a clinically approved monoclonal antibody against the IL-6 receptor, Tocilizumab, was tested in 14 COVID-19-S patients in Anhui Hospital affiliated with the University of Science and Technology of China [47]. A preliminary study indicated that a high body temperature of 11 out of the 14 COVID-19 patients was reduced to normal within 24 hours of Tocilizumab treatment and the lung-injury syndromes were markedly alleviated [47]. A clinical trial of Tocilizumab therapy in COVID-19 patients was registered and is now ongoing.

In summary, it is critical to identify the functions of specific, elevated cytokines and evaluate the potential consequences of treatment before considering modulating therapy, otherwise it may exacerbate the disease conditions. Our data suggest that modulators of elevated cytokines/chemokines may provide potential precision medical treatments for the current pandemic of 2019-nCoV.

## METHODS

### Experimental ethics policy

The study protocol was approved by the Ethics Committees of Shenzhen Third People's Hospital (SZTHEC2016001). Verbal informed consent was obtained from all patients or family members of patients with 2019-nCoV and H7N9 infections due to the special circumstances that pens and paper were not allowed to be brought into containment facilities. The informed consent from bacteria-infected patients and healthy individuals was obtained by normal procedures. The study was conducted in accordance with the International Conference on Harmonisation Guidelines for Good Clinical Practice and the Declaration of Helsinki and institutional ethics guidelines.

### Acquisition of clinical specimens

Throat swabs, rectal swabs, and sputum and BALF specimens were collected within 24 hours of blood-sample collection from laboratory-confirmed COVID-19 cases upon admission in January 2020 and at various time points thereafter. Plasma samples were collected from eight healthy subjects undergoing wellness examinations in the hospital in the interim. In addition, we obtained archived plasma samples from eight patients with laboratory-confirmed H7N9 infections who were hospitalized between January 2015 and March 2017 and eight bacterial pneumonia patients who were hospitalized between August and December 2019. The archived blood samples were stored at –80°C since collection.

### qRT-PCR

Viral RNAs were extracted from 140 μl of clinical specimens within 6 hours of sample collection using the QIAamp RNA Viral Kit (Qiagen, Heiden, Germany) as instructed by the manufacturer. Then, 2 μl extracted viral RNAs were amplified by quantitative reverse transcription polymerase chain reaction (qRT-PCR) using primers and probes recommended by Chinese Center for Diseases Control and Prevention (China CDC) [48] and a commercially available kit for 2019-nCoV detection (GeneoDX Co., Shanghai, China). Quantitative analyses of the 2019-nCoV viral load of clinical specimens were shown as a cycle threshold (C_t_) value—an intrinsic characteristic of qRT-PCR that provided a semi-quantitative measure of viral load, in that higher virus loads were reflected by lower C_t_ values [30, 49]. Samples with a C_t_ value ≤37.0 were considered putatively positive. Samples whose C_t_ was higher than 37 were retested and considered positive if C_t_ was ≥37 but ≤40 and negative if viral RNAs were undetectable on the second test.

### Disease-severity classification and Murray-score calculation

The severity of 2019-nCoV infection was graded according to the China National Health Commission Guidelines for Diagnosis and Treatment of 2019-nCoV infection. Briefly, a patient was considered to have mild COVID-19 pneumonia if he or she had fever, respiratory manifestations and radiological findings indicative of pneumonia. A patient was considered to have severe COVID-19 pneumonia if he or she met any of the following: (i) respiratory distraction (respiration rate ≥30/min); (ii) resting oxygen saturation ≤93% or (iii) arterial oxygen partial pressure (PaO_2_)/fraction of inspired oxygen (FiO_2_) ≤300 mmHg (1 mmHg = 0.133 kPa). A patient was considered to have critical COVID-19 pneumonia if he or she had any of the following: (i) respiratory failure requiring mechanical ventilation, (ii) shock or (iii) failure of other organs requiring intensive-care-unit care. Clinicopathological variables of 12 patients with 2019-nCoV infections including 4 COVID-19-M patients, 5 COVID-19-S patients and 3 COVID-19-C patients were collected at admission and the disease severity was assessed using Murray scores [50].

### Cytokine and chemokine measurements

Plasma concentrations of 48 cytokines and chemokines were measured in duplicate using Bio-Plex Pro Human Cytokine Screening Panel (48-Plex#12007283, Bio-Rad) according to the manufacturer's instructions. All the plasma samples were fixed in 2% paraformaldehyde at the same time before analysis and measured in a biosafety level III laboratory.

### Statistical analysis

The Spearman’s rank correlation coefficient was used for linear-correlation analysis between plasma cytokine levels and viral load (throat swabs, sputum and BALF) and the Murray score of patients with 2019-nCoV infections. The ROC AUC of plasma cytokine levels was estimated for COVID-19-M and COVID-19-S infections. ANOVA or Mann–Whitney U-test were used to compare plasma cytokine levels among the groups. All statistical tests were calculated using SPSS 16.0 for Windows (SPSS, Inc., Chicago, IL, USA). A two-tailed *P*-value < 0.05 was considered to be statistically significant.

## Supplementary Material

nwaa037_Supplemental_Files
