# Supplementary material for: Elevated plasma levels of selective cytokines in COVID-19 patients reflect viral load and lung injury
Source: Natl Sci Rev. 2020 Mar 9;7(6):1003–11. doi: 10.1093/nsr/nwaa037 (PMC7107806; doi:10.1093/nsr/nwaa037)
Supplement: nwaa037_Supplemental_Files [file nwaa037_supplemental_files.pdf]

Figure 1

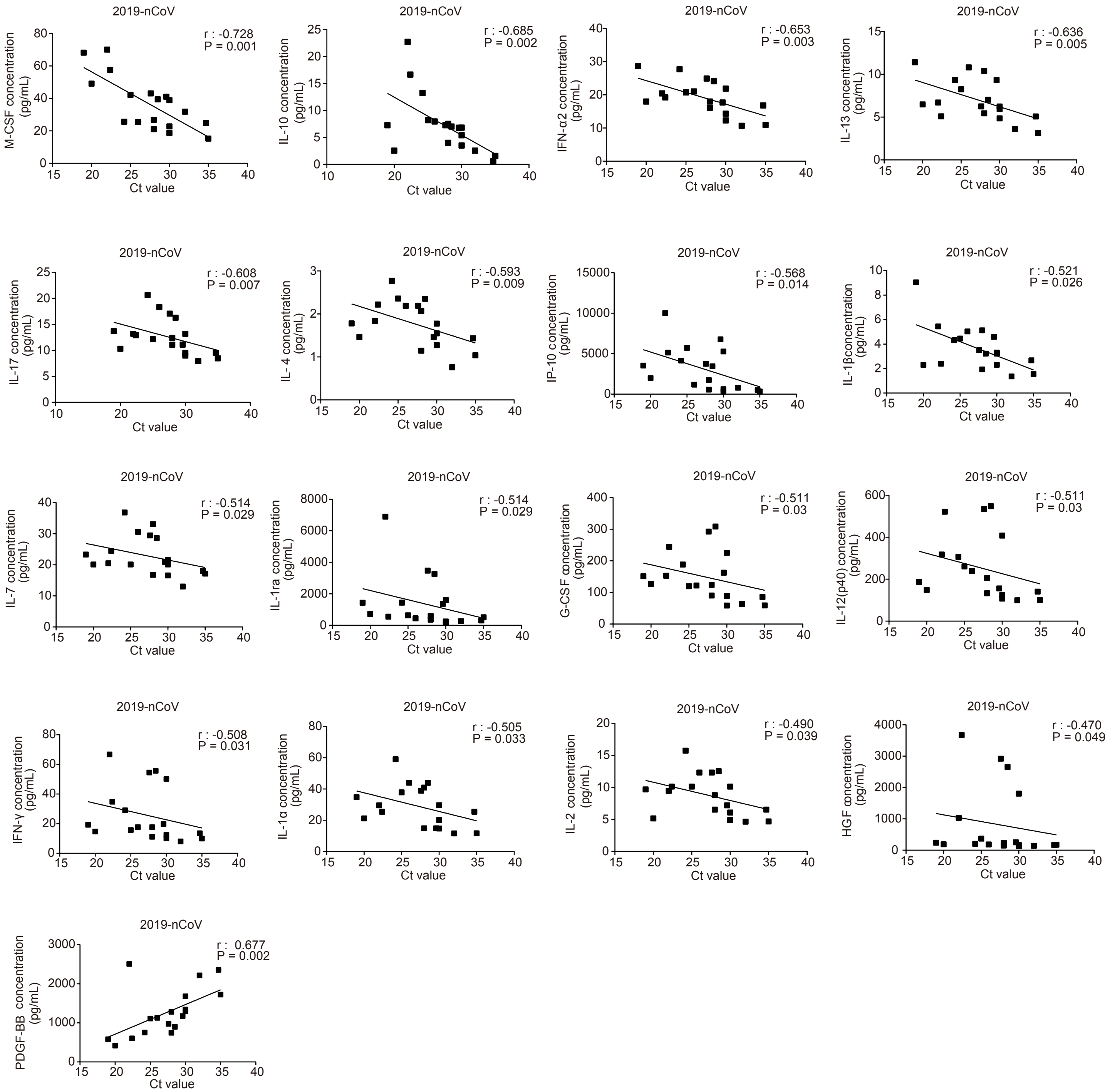

2019-nCoV
M-CSF concentration (pg/mL)
r : -0.728
P = 0.001
Ct value
2019-nCoV
IL-10 concentration (pg/mL)
r : -0.685
P = 0.002
Ct value
2019-nCoV
IFN-α2 concentration (pg/mL)
r : -0.653
P = 0.003
Ct value
2019-nCoV
IL-13 concentration (pg/mL)
r : -0.636
P = 0.005
Ct value
2019-nCoV
IL-17 concentration (pg/mL)
r : -0.608
P = 0.007
Ct value
2019-nCoV
IL- 4 concentration (pg/mL)
r : -0.593
P = 0.009
Ct value
2019-nCoV
IP-10 concentration (pg/mL)
r : -0.568
P = 0.014
Ct value
2019-nCoV
IL-1βconcentration (pg/mL)
r : -0.521
P = 0.026
Ct value
2019-nCoV
IL-7 concentration (pg/mL)
r : -0.514
P = 0.029
Ct value
2019-nCoV
IL-1ra concentration (pg/mL)
r : -0.514
P = 0.029
Ct value
2019-nCoV
G-CSF concentration (pg/mL)
r : -0.511
P = 0.03
Ct value
2019-nCoV
IL-12(p40) concentration (pg/mL)
r : -0.511
P = 0.03
Ct value
2019-nCoV
IFN-γ concentration (pg/mL)
r : -0.508
P = 0.031
Ct value
2019-nCoV
IL-1α concentration (pg/mL)
r : -0.505
P = 0.033
Ct value
2019-nCoV
IL-2 concentration (pg/mL)
r : -0.490
P = 0.039
Ct value
2019-nCoV
HGF concentration (pg/mL)
r : -0.470
P = 0.049
Ct value
2019-nCoV
PDGF-BB concentration (pg/mL)
r : 0.677
P = 0.002
Ct value

Figure. 2

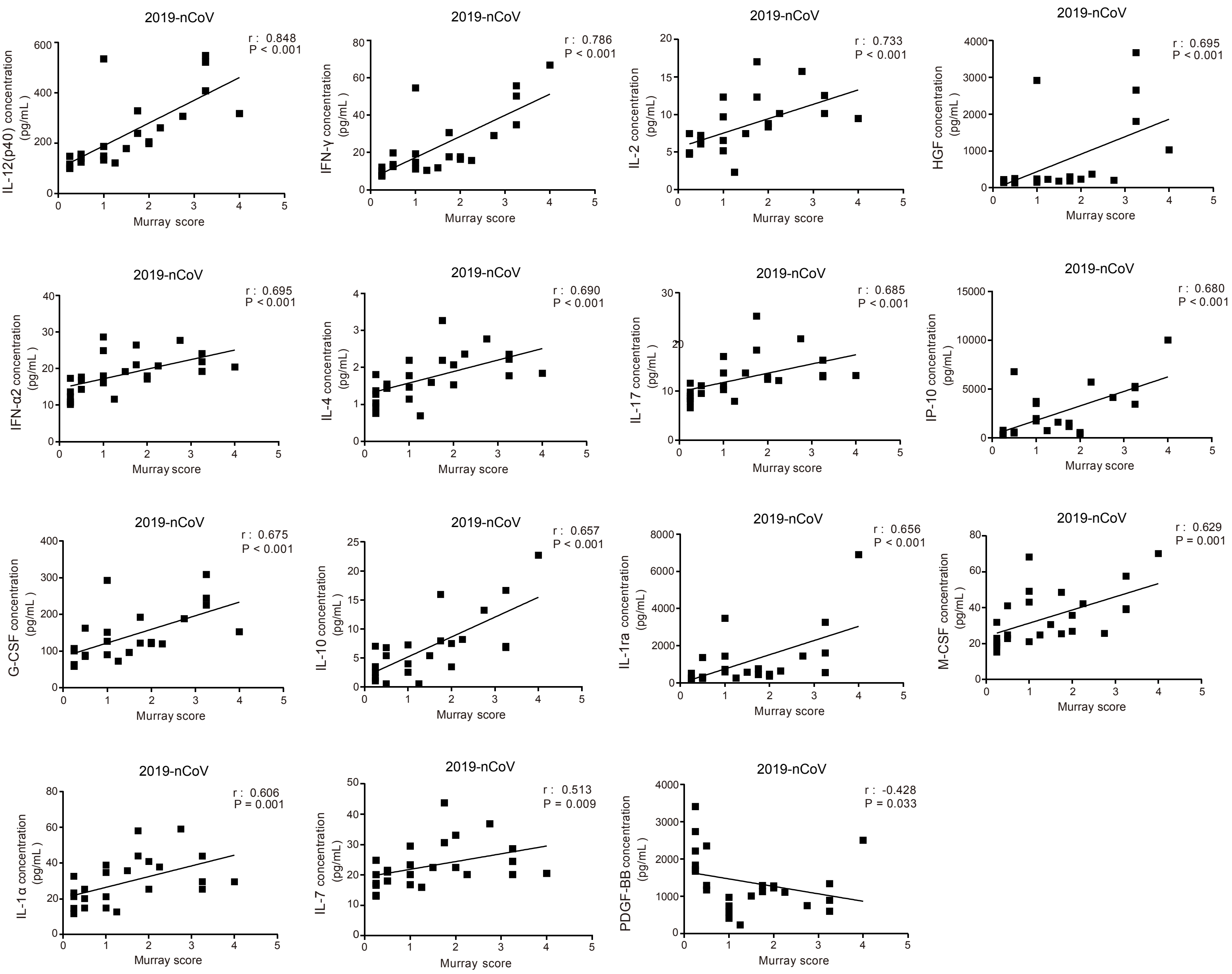
2019-nCoV
r : 0.848
P < 0.001
IL-12(p40) concentration (pg/mL)
Murray score
2019-nCoV
r : 0.786
P < 0.001
IFN-γ concentration (pg/mL)
Murray score
2019-nCoV
r : 0.733
P < 0.001
IL-2 concentration (pg/mL)
Murray score
2019-nCoV
r : 0.695
P < 0.001
HGF concentration (pg/mL)
Murray score
2019-nCoV
r : 0.695
P < 0.001
IFN-α2 concentration (pg/mL)
Murray score
2019-nCoV
r : 0.690
P < 0.001
IL-4 concentration (pg/mL)
Murray score
2019-nCoV
r : 0.685
P < 0.001
IL-17 concentration (pg/mL)
Murray score
2019-nCoV
r : 0.680
P < 0.001
IP-10 concentration (pg/mL)
Murray score
2019-nCoV
r : 0.675
P < 0.001
G-CSF concentration (pg/mL)
Murray score
2019-nCoV
r : 0.657
P < 0.001
IL-10 concentration (pg/mL)
Murray score
2019-nCoV
r : 0.656
P < 0.001
IL-1ra concentration (pg/mL)
Murray score
2019-nCoV
r : 0.629
P = 0.001
M-CSF concentration (pg/mL)
Murray score
2019-nCoV
r : 0.606
P = 0.001
IL-1α concentration (pg/mL)
Murray score
2019-nCoV
r : 0.513
P = 0.009
IL-7 concentration (pg/mL)
Murray score
2019-nCoV
r : -0.428
P = 0.033
PDGF-BB concentration (pg/mL)
Murray score

Figure 3

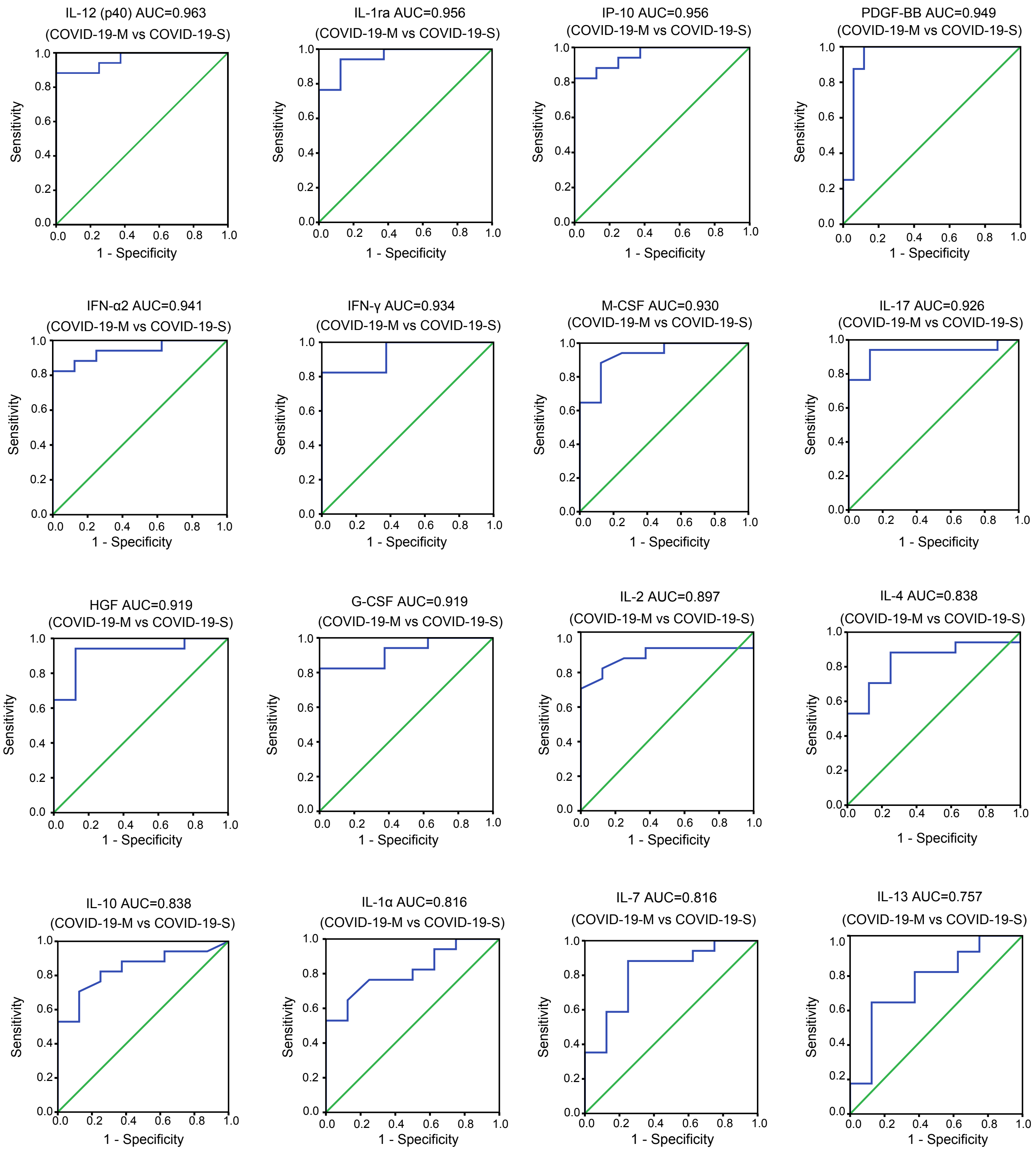
IL-12 (p40) AUC=0.963
(COVID-19-M vs COVID-19-S)
IL-1ra AUC=0.956
(COVID-19-M vs COVID-19-S)
IP-10 AUC=0.956
(COVID-19-M vs COVID-19-S)
PDGF-BB AUC=0.949
(COVID-19-M vs COVID-19-S)
IFN-α2 AUC=0.941
(COVID-19-M vs COVID-19-S)
IFN-γ AUC=0.934
(COVID-19-M vs COVID-19-S)
M-CSF AUC=0.930
(COVID-19-M vs COVID-19-S)
IL-17 AUC=0.926
(COVID-19-M vs COVID-19-S)
HGF AUC=0.919
(COVID-19-M vs COVID-19-S)
G-CSF AUC=0.919
(COVID-19-M vs COVID-19-S)
IL-2 AUC=0.897
(COVID-19-M vs COVID-19-S)
IL-4 AUC=0.838
(COVID-19-M vs COVID-19-S)
IL-10 AUC=0.838
(COVID-19-M vs COVID-19-S)
IL-1α AUC=0.816
(COVID-19-M vs COVID-19-S)
IL-7 AUC=0.816
(COVID-19-M vs COVID-19-S)
IL-13 AUC=0.757
(COVID-19-M vs COVID-19-S)
Sensitivity
1 - Specificity
0.0
0.2
0.4
0.6
0.8
1.0

Figure. 4

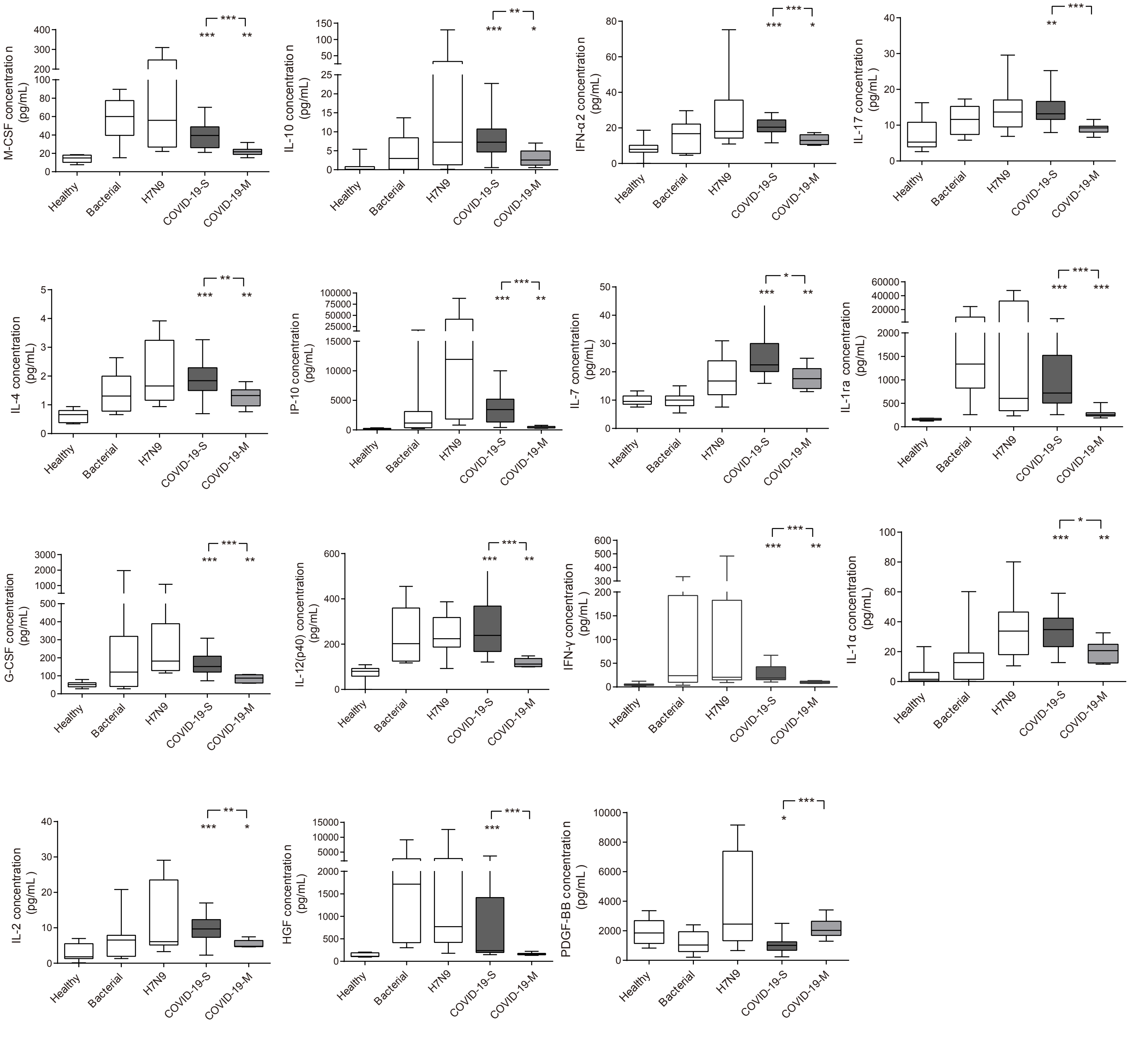

M-CSF concentration (pg/mL)
IL-10 concentration (pg/mL)
IFN-α2 concentration (pg/mL)
IL-17 concentration (pg/mL)
IL-4 concentration (pg/mL)
IP-10 concentration (pg/mL)
IL-7 concentration (pg/mL)
IL-1ra concentration (pg/mL)
G-CSF concentration (pg/mL)
IL-12(p40) concentration (pg/mL)
IFN-γ concentration (pg/mL)
IL-1α concentration (pg/mL)
IL-2 concentration (pg/mL)
HGF concentration (pg/mL)
PDGF-BB concentration (pg/mL)
Healthy
Bacterial
H7N9
COVID-19-S
COVID-19-M

Table S1. Epidemiological and clinical features of subjects hospitalized with 2019-nCoV, H7N9 avian influenza virus and bacterial infections.

Characteristics	2019-nCoV	H7N9	Bacteria	Control
**Median age (range)**	62.5 (10-72)	56 (21-67)	41 (31.5-49)	28 (25-34)
**Age subgroups**				
0–15 years	1/12 (8.3%)	0/8 (0%)	1/8 (12.5%)	0/8 (0%)
16–59 years	4/12 (33.3%)	6/8 (75%)	5/8 (62.5%)	8/8 (100%)
≥60 years	7/12 (58.3%)	2/8 (25%)	2/8 (25%)	0/8 (0%)
**Male (%)**	8/12 (66.7%)	5/8 (62.5%)	5/8 (62.5%)	4/8 (50%)
**Initial symptoms**				
Fever	9/12 (75%)	8/8 100%)	5/8 (62.5%)	0/8 (0%)
Cough	12/12 (100%)	7/8 (75%)	3/8 (37.5%)	0/8 (0%)
Headache	0/12 (0%)	3/8 (37.5%)	1/8 (12.5%)	0/8 (0%)
Myalgia	4/12 (33.3%)	4/8 (50%)	0/8 (0%)	0/8 (0%)
Chill	5/12 (41.7%)	5/8 (62.5%)	0/8 (0%)	0/8 (0%)
Nausea or vomiting	2/12 (16.7%)	0/8 (0%)	1/8 (12.5%)	0/8 (0%)
Diarrhea	3/12 (25%)	2/8 (25%)	0/8 (0%)	0/8 (0%)
**Co-existing chronic medical conditions**	6/12 (50%)	6/8 (50%)	5/8 (62.5%)	0/8 (0%)
Chronic heart disease	4/12 (33.3%)	4/8 (33.3%)	2/8 (25%)	0/8 (0%)
Chronic lung disease	1/12 (8.3%)	5/8 (41.7%)	1/8 (12.5%)	0/8 (0%)
Chronic renal disease	2/12 (16.7%)	2/8(16.7%)	0/8 (0%)	0/8 (0%)
Chronic liver disease	1/12 (8.3%)	3/8 (25%)	0/8 (0%)	0/8 (0%)
Diabetes	2/12 (16.7%)	6/8 (50%)	0/8 (0%)	0/8 (0%)
Cancer	0/12 (0%)	4/8 (33.3%)	1/8 (12.5%)	0/8 (0%)
**Bacterial co-infections**	2/12 (16.7%)	7/8 (87.5%)	NA	0/8 (0%)
**Interval, median days (IQ+R)**				
Onset to admission	6.5 (5, 9.25)	6 (4.75, 7)	1 (0, 3.5)	NA
Onset to starting antiviral treatment	6 (5, 9.25)	5.5 (3.75, 6.25)	NA	NA
Onset to laboratory confirmation	7 (4.5, 14.5)	7 (5.75, 8.25)	6 (4.5, 8)	NA
**Complications**				
Pneumonia	12/12 (100%)	8/8 (100%)	8/8 (100%)	0/8 (0%)
ARDS	6/12 (50%)	7/8 (87.5%)	6/8 (75%)	0/8 (0%)
SevereARDS	2/12 (16.7%)	5/8 (62.5%)	2/8 (25%)	0/8 (0%)
Respiratory failure	3/12 (25%)	7/8 (87.5%)	5/8 (62.5%)	0/8 (0%)
Hepatic insufficiency	2/12 (16.7%)	4/8 (50%)	4/8 (50%)	0/8 (0%)
Renal insufficiency	2/12 (16.7%)	2/8 (25%)	5/8 (62.5%)	0/8 (0%)
Cardiac failure	1/12 (8.3%)	3/8 (37.5%)	3/8 (37.5%)	0/8 (0%)
Shock	1/12 (8.3%)	1/8 (12.5%)	4/8 (50%)	0/8 (0%)
**Treatment**				
Received antivirals≤ 2 days after illness onset	1/12 (8.3%)	1/8 (12.5%)	NA	NA
Received antivirals 3-5 days after illness onset	4/12 (33.3%)	3/8 (37.5%)	NA	NA
Received antivirals 6 days after illness onset	7/12 (58.3%)	4/8 (50%)	NA	NA
Corticosteroid	2/12 (16.7%)	8/8 (100%)	2/8 (25%)	NA
Mechanical ventilation	5/12 (41.7%)	7/8 (87.5%)	7/8 (87.5%)	NA

NA: Not applicable.

Table S2. Cytokine comparison among healthy controls, bacteria-infected patients, H7N9-infected patients and 2019-nCoV-infected patients (disease severity classified)

Cytokine	Healthy						Bacteria					H7N9				2019-nCoV				
	Mean	SE	P^a value	P^b value	P^c value	P^d value	Mean	SE	P^e value	P^f value	P^g value	Mean	SE	P^h value	P^i value	COVID-19-M		COVID-19-S		P^j value
																Mean	SE	Mean	SE	
IL-1β	1.64	0.51	#	0.002	-	0.002	3.23	0.76	0.040	-	-	6.74	1.34	0.036	#	3.10	0.71	4.12	0.42	-
IL-1ra	156.74	7.40	0.001	0.001	<0.001	<0.001	5576.32	2956.33	-	0.002	-	11882.61	7235.11	0.016	-	283.15	35.42	1460.46	409.56	<0.001
IL-2	2.90	0.88	#	0.020	0.045	<0.001	6.89	2.21	-	-	0.041	12.16	3.64	-	-	5.46	0.38	9.71	0.88	0.002
IL-4	0.63	0.08	0.010	0.001	0.003	<0.001	1.43	0.25	-	-	#	2.03	0.41	-	-	1.27	0.12	1.92	0.15	0.007
IL-5	0.10	0.00	-	#	-	-	5.33	5.23	-	-	-	19.36	9.92	#	0.008	0.10	0.00	0.10	0.00	-
IL-6	4.87	1.68	0.006	-	-	0.004	201.55	109.57	-	0.002	-	199.98	130.85	#	-	3.26	0.60	22.79	6.93	<0.001
IL-7	10.01	0.67	-	0.020	0.002	<0.001	10.08	1.01	0.018	0.002	<0.001	17.84	2.67	-	#	18.05	1.42	25.26	1.81	0.012
IL-8	3.90	0.61	0.002	0.002	0.013	<0.001	74.98	32.66	-	0.021	-	102.02	53.69	0.006	-	6.53	0.55	18.80	2.84	0.001
IL-9	232.22	14.53	-	0.027	#	-	220.64	10.09	0.007	0.006	0.031	286.58	17.77	-	#	264.29	4.16	248.00	4.63	0.020
IL-10	0.89	0.66	-	0.009	0.011	<0.001	4.43	1.84	-	-	0.070	24.76	15.64	-	-	3.03	0.78	8.44	1.37	0.007
IL-12 (p70)	2.64	0.87	-	-	-	0.010	2.33	0.68	-	-	0.005	8.36	4.90	-	-	3.92	0.72	6.78	0.93	0.027
IL-13	2.73	0.66	-	0.026	0.008	<0.001	2.49	0.33	0.020	0.002	<0.001	8.49	2.44	-	-	5.98	0.80	8.01	0.52	0.041
IL-15	217.59	94.07	-	-	-	-	157.08	66.09	-	-	-	423.26	187.32	-	-	202.93	51.64	189.16	36.19	-
IL-17	7.12	1.66	0.045	0.015	-	0.003	11.59	1.55	-	-	-	14.69	2.46	0.036	-	9.06	0.52	14.24	1.02	<0.001
Eotaxin	30.68	5.20	-	-	-	#	41.14	10.93	-	-	-	56.77	13.86	-	-	37.23	2.56	44.67	4.74	-
FGF basic	30.80	1.82	-	0.020	-	<0.001	42.67	5.32	-	-	-	64.96	18.07	-	-	35.60	2.74	44.40	1.94	0.020
G-CSF	51.61	5.66	-	0.001	0.009	<0.001	367.40	232.15	-	-	-	321.14	114.59	<0.001	-	83.99	7.42	164.24	16.74	<0.001
GM-CSF	1.31	0.54	-	#	#	0.020	1.61	0.54	-	-	0.036	7.99	4.11	-	-	2.36	0.30	3.28	0.41	-
IFN-γ	5.21	1.12	0.010	0.001	0.006	<0.001	86.89	44.55	-	#	-	104.98	60.00	0.005	-	10.14	0.84	28.00	4.37	<0.001
IP-10	192.09	29.31	0.002	0.001	0.002	<0.001	3293.89	2007.72	0.046	-	-	22962.93	10848.70	<0.001	#	453.41	60.78	3386.70	631.17	<0.001
MCP-1 (MCAF)	8.78	1.91	#	0.024	0.036	<0.001	354.66	221.30	-	-	-	265.58	209.79	-	-	16.25	2.81	64.83	13.83	0.004
MIP-1α	1.32	0.09	0.008	0.001	0.005	<0.001	6.73	2.46	-	-	-	5.68	1.58	0.005	-	2.21	0.29	4.35	0.77	0.014
PDGF-BB	1972.26	305.97	#	-	-	0.010	1192.82	276.37	0.036	0.036	-	3675.14	1199.46	-	0.006	2157.76	239.56	1017.38	122.07	<0.001
MIP-1β	167.91	15.86	-	-	-	-	158.80	13.59	#	#	-	184.35	13.52	-	-	167.82	3.48	158.13	4.06	#
RANTES	4993.07	1385.58	0.046	0.027	0.021	-	7402.33	2024.74	-	-	#	14094.99	4181.73	-	0.012	6699.77	661.12	4677.52	494.74	0.014
TNF-α	53.19	4.47	0.036	0.006	0.036	<0.001	72.20	6.84	-	-	-	118.23	24.93	#	-	69.36	1.20	82.58	3.83	0.048
VEGF	105.72	27.11	-	0.046	-	-	114.11	24.96	0.045	-	-	265.98	76.46	0.036	0.027	108.66	9.21	130.88	10.77	#
IL-1α	5.05	2.75	-	0.002	0.005	<0.001	15.89	6.91	0.045	-	0.012	35.82	7.71	#	-	20.11	2.57	33.38	3.34	0.012
IL-2Rα	53.32	6.15	0.020	0.002	-	0.004	160.43	36.94	-	0.021	-	193.98	40.71	0.003	0.020	61.41	7.01	94.64	9.83	0.041
IL-3	0.23	0.08	-	#	#	0.003	0.33	0.14	-	-	0.006	1.38	0.82	-	-	0.44	0.10	0.74	0.10	0.044
IL-12 (p40)	71.51	11.89	0.001	0.002	0.003	<0.001	383.78	137.78	-	0.005	-	376.17	139.09	0.012	-	117.36	6.69	282.06	34.79	<0.001
IL-16	19.92	11.27	0.015	0.035	#	#	321.46	155.90	-	-	-	351.56	176.31	-	-	91.35	42.02	61.56	18.03	-
IL-18	28.90	7.51	0.002	0.002	0.005	<0.001	305.77	82.64	-	0.012	0.041	541.14	290.27	0.027	0.048	69.90	5.79	101.17	12.93	-
CTACK	310.34	76.56	0.005	0.003	0.021	0.005	1469.44	451.98	-	-	#	1154.40	252.81	#	0.041	558.71	64.27	653.19	85.09	-
GRO-α	937.91	160.22	-	-	-	-	610.87	39.31	-	0.016	-	786.16	99.14	-	-	730.27	25.44	652.08	18.89	#
HGF	137.42	15.75	0.001	0.002	-	<0.001	2375.54	1030.70	-	<0.001	0.023	2548.55	1476.82	0.001	-	166.35	9.81	872.79	278.63	<0.001
IFN-α2	8.30	1.84	-	0.004	0.027	<0.001	15.69	3.26	-	-	-	26.71	7.63	0.036	-	13.28	0.97	20.76	1.09	<0.001
LIF	0.10	0.00	0.011	0.027	0.027	0.001	29.48	16.43	-	-	-	64.48	27.74	-	-	18.25	8.25	23.87	5.79	-
MCP-3	1.78	0.89	-	0.004	#	<0.001	20.95	11.03	-	-	-	19.09	13.20	0.011	-	2.64	0.40	8.73	1.19	<0.001
M-CSF	14.30	1.47	0.003	0.001	0.003	<0.001	57.27	8.43	-	0.012	#	113.37	42.15	0.006	-	22.06	1.75	40.49	3.57	<0.001
MIF	1095.90	372.60	0.012	0.021	0.036	-	2466.29	664.87	-	-	0.017	4505.83	1729.28	-	#	1438.12	177.56	1153.54	117.92	-
MIG	63.20	12.69	0.010	0.001	0.016	<0.001	622.95	211.23	0.046	0.021	-	18787.15	13163.69	0.002	0.036	130.85	29.20	1088.53	315.64	0.003
b-NGF	0.64	0.24	#	0.006	0.013	<0.001	3.06	1.36	-	-	-	4.57	2.20	0.027	-	1.54	0.14	2.63	0.34	0.009
SCF	44.53	8.44	0.024	0.006	-	0.002	153.21	43.95	-	-	-	222.52	120.66	0.016	-	49.18	4.13	85.73	9.64	0.004
SCGF-β	48340.78	5710.60	0.002	0.001	#	0.006	140757.32	28033.76	-	0.009	-	176450.97	29405.62	0.002	0.012	64805.66	6401.71	99512.14	12073.24	-
SDF-1α	693.52	98.80	0.036	-	-	0.048	907.92	85.68	-	-	-	931.76	119.70	-	-	760.08	50.90	780.15	33.18	-
TNF-β	0.10	0.00	-	0.004	0.010	0.001	1.49	1.02	0.033	-	-	6.29	1.82	-	-	1.27	0.47	2.38	0.47	-
TRAIL	27.86	1.73	-	0.014	0.002	<0.001	32.92	3.90	-	-	0.027	58.34	20.27	-	-	37.62	1.50	43.87	2.16	#

P^a value: Healthy vs bacteria patients
P^b value: Healthy vs H7N9 patients
P^c value: Healthy vs COVID-19-M patients
P^d value: Healthy vs COVID-19-S patients
P^e value: Bacteria patients vs H7N9 patients
P^f value: Bacteria patients vs COVID-19-M patients
P^g value: Bacteria patients vs COVID-19-S patients
P^h value: H7N9 patients vs COVID-19-M patients
P^i value: H7N9 patients vs COVID-19-S patients
P^j value : COVID-19-M patients vs COVID-19-S patients
#：P value [0.05,0.1), -：P value >0.05

8 samples from 8 healthy control, 8 samples from 8 bacteria-infected patients, 8 samples from 8 H7N9-infected patients, 8 samples from 4 mild 2019-nCoV-infected patients (COVID-19-M) and 17 samples from 8 severe 2019-nCoV infected patients (COVID-19-S).

Table S3. Cytokine comparison among healthy controls, bacteria-infected patients, H7N9-infected patients and 2019-nCoV infected patients (Day0-7, Day8-14, Day15-)

Cytokine	Healthy			Bacteria			H7N9			2019-nCoV								
	P^a value	P^b value	P^c value	P^d value	P^e value	P^f value	P^g value	P^h value	P^i value	Day 0-7 Mean	Day 0-7 SE	Day 8-14 Mean	Day 8-14 SE	Day 15- Mean	Day 15- SE	P^j value	P^k value	P^l value
IL-1β	0.037	0.020	0.019	-	-	-	-	0.046	#	4.08	1.02	3.63	0.48	3.35	0.49	-	-	-
IL-1ra	0.001	<0.001	0.003	#	0.011	-	-	-	-	1478.23	917.46	889.43	308.43	867.02	259.33	-	-	-
IL-2	0.027	0.002	0.005	-	-	-	-	-	-	6.44	0.84	8.91	1.12	7.74	0.68	-	#	-
IL-4	0.004	0.001	0.003	-	-	-	-	-	-	1.32	0.15	1.90	0.18	1.47	0.10	0.043	-	-
IL-5	-	-	-	-	-	-	#	0.020	-	0.10	0.00	0.10	0.00	0.10	0.00	-	-	-
IL-6	-	#	#	0.028	0.017	-	-	-	-	9.98	3.54	10.07	2.22	34.62	22.56	-	#	-
IL-7	0.003	0.001	0.003	0.003	<0.001	0.003	-	#	-	17.66	1.46	25.54	2.10	19.65	1.02	0.017	-	-
IL-8	0.008	0.001	0.003	#	-	-	0.049	-	-	9.87	2.79	15.85	3.24	16.70	6.49	-	-	-
IL-9	-	-	-	0.049	0.030	0.019	-	-	-	254.65	6.41	248.48	5.69	263.36	5.75	-	-	-
IL-10	0.006	0.001	0.009	-	-	-	-	-	-	6.45	2.79	7.05	1.36	4.33	1.17	-	-	-
IL-12 (p70)	-	0.014	0.040	-	0.012	0.012	-	-	-	3.62	0.34	6.74	1.12	4.78	0.53	-	0.034	-
IL-13	0.011	0.001	0.008	0.002	<0.001	0.003	-	-	-	6.61	0.90	7.65	0.67	6.86	1.06	-	-	-
IL-15	-	-	-	-	-	-	-	-	-	104.65	43.18	218.73	39.35	190.10	66.90	#	-	-
IL-17	#	0.008	0.040	-	-	-	-	-	-	10.26	0.99	13.49	1.33	11.56	0.67	-	-	-
Eotaxin	#	-	-	-	-	-	-	-	-	44.86	6.72	38.29	4.69	45.29	8.15	-	-	-
FGF basic	-	0.003	0.008	-	-	-	-	-	-	37.23	3.52	43.14	2.51	39.63	3.85	-	-	-
G-CSF	0.005	0.001	0.003	-	-	-	#	0.036	-	107.31	14.62	148.44	23.14	136.99	26.02	-	-	-
GM-CSF	#	0.038	#	-	#	#	-	-	-	2.43	0.36	3.15	0.48	2.37	0.38	-	-	-
IFN-γ	0.005	0.001	0.003	-	-	-	-	-	-	19.65	8.00	22.36	4.59	22.20	7.14	-	-	-
IP-10	0.002	<0.001	0.003	-	-	-	#	0.007	#	2680.72	1294.44	1946.64	529.30	2944.85	1304.24	-	-	-
MCP-1 (MCAF)	0.028	0.001	0.008	-	-	-	-	-	-	34.48	11.44	41.98	10.99	74.12	40.87	-	-	-
MIP-1α	0.009	<0.001	0.004	-	-	-	0.049	-	-	2.54	0.35	4.22	1.00	3.63	0.98	-	-	-
PDGF-BB	-	#	-	-	-	-	-	0.025	-	1651.44	426.27	1241.39	170.50	1370.23	266.40	-	-	-
MIP-1β	-	-	-	-	-	-	-	-	-	160.45	3.18	159.07	5.19	169.04	5.34	-	-	-
RANTES	-	-	-	-	-	-	#	0.043	-	5683.14	925.79	4763.56	456.24	6404.49	1272.99	-	-	-
TNF-α	#	0.002	0.003	-	-	#	-	-	-	70.85	4.36	81.14	4.78	76.86	4.51	-	0.042	-
VEGF	-	-	-	-	-	-	#	0.030	#	112.19	12.95	126.58	12.13	113.28	9.97	-	-	-
IL-1α	0.006	<0.001	0.005	-	0.017	#	-	-	-	21.46	3.21	32.58	3.78	22.07	3.02	#	-	-
IL-2Rα	-	0.014	#	#	-	-	0.008	0.017	#	68.48	11.31	91.07	12.53	89.23	10.26	-	-	-
IL-3	-	0.003	#	-	0.014	0.039	-	-	-	0.38	0.05	0.77	0.11	0.45	0.05	0.017	-	-
IL-12 (p40)	0.004	<0.001	0.003	0.049	-	-	-	-	-	156.36	29.38	263.82	46.56	207.24	51.43	-	#	-
IL-16	0.049	-	0.035	-	-	-	-	-	-	56.54	15.36	48.52	18.26	154.14	65.84	-	-	-
IL-18	0.004	0.001	0.008	#	0.017	-	-	0.025	-	111.64	28.12	79.63	8.04	95.26	12.86	-	-	-
CTACK	#	0.006	0.019	-	-	-	#	#	-	496.00	83.70	641.32	88.69	728.05	174.99	-	-	-
GRO-α	-	-	-	-	-	0.040	-	-	-	669.95	20.46	657.17	23.28	723.07	51.58	-	-	-
HGF	0.037	0.006	0.013	0.005	0.025	0.019	0.028	#	#	305.64	121.51	875.59	354.58	518.05	322.31	-	-	-
IFN-α2	0.015	0.001	0.012	-	-	-	-	-	-	16.63	2.47	18.58	1.37	17.93	1.02	-	-	-
LIF	0.047	0.001	0.018	-	-	-	-	-	-	12.58	7.97	28.11	6.11	5.77	4.54	#	-	-
MCP-3	0.036	0.003	0.012	-	-	-	-	-	-	4.43	1.35	7.29	1.35	6.81	2.79	-	#	-
M-CSF	0.001	0.001	0.003	-	0.017	#	-	#	-	39.97	8.50	32.22	3.53	32.31	3.97	-	-	-
MIF	#	-	-	-	0.020	-	-	#	-	1295.66	173.44	1116.72	113.22	1511.60	325.36	-	-	-
MIG	0.011	0.001	0.005	-	-	-	0.021	0.020	#	469.21	258.66	939.15	390.93	749.79	489.35	-	-	-
b-NGF	0.017	0.002	0.005	-	-	-	-	-	-	1.49	0.18	2.55	0.42	2.04	0.25	0.047	0.015	-
SCF	-	0.007	0.019	-	-	-	-	-	-	58.44	8.34	78.61	12.45	78.51	16.59	-	-	-
SCGF-β	0.037	0.017	#	-	#	-	0.028	0.005	0.040	84399.44	14867.23	93153.33	14483.77	78223.91	18421.01	-	-	-
SDF-1α	-	-	#	-	-	-	-	-	-	761.70	52.72	750.62	33.61	846.56	78.83	-	-	-
TNF-β	-	0.001	0.001	-	#	#	#	-	-	0.91	0.57	2.35	0.51	1.62	0.52	#	#	-
TRAIL	0.002	0.001	0.003	#	#	-	-	-	-	43.32	3.81	39.70	1.77	45.13	4.31	-	-	-

P^a value: Healthy vs COVID-19 patients (Day0-7)
P^b value: Healthy vs COVID-19 patients (Day8-14)
P^c value: Healthy vs COVID-19 patients (Day15-)
#：P value [0.05,0.1), -：P value >0.05

P^d value: Bacteria patients vs COVID-19 patients (Day0-7)
P^e value: Bacteria patients vs COVID-19 patients (Day8-14)
P^f value: Bacteria patients vs COVID-19 patients (Day15-)

P^g value: H7N9 patients vs COVID-19 patients (Day0-7)
P^h value: H7N9 patients vs COVID-19 patients (Day8-14)
P^i value: H7N9 patients vs COVID-19 patients (Day15-)

P^j value：COVID-19 patients (Day0-7) vs COVID-19 patients (Day8-14)
P^k value：COVID-19 patients (Day0-7) vs COVID-19 patients (Day15-)
P^l value：COVID-19 patients (Day8-14) vs COVID-19 patients (Day15-)

8 samples from 8 healthy control, 8 samples from 8 bacteria-infected patients, 8 samples from 8 H7N9-infected patients, 7 samples from 7 2019-nCoV-infected patients (Day0-7), 13 samples from 9 2019-nCoV-infected patients (Day8-14) and 5 samples from 5 2019-nCoV-infected patients (Day15-).

Table S4. Detection of 2019-nCoV at respiratory and non-respiratory sites.

		2019-nCoV		
		Total	Severe	Mild
**Nasopharynx**				
Detectable RNA (n/N)	Throat	8/11	5/7	3/4
	Sputum	7/10	6/6	1/4
Ct Values (Median; range)	Throat	30（23-36）	29.2 (25-36)	30 (23-35)
	Sputum	29.1 (20-34.7)	28.3 (20-33.4)	34.3
**BALF**				
Detectable RNA (n/N)		3/5	3/4	0/1
Ct Values (Median; range)		24 (19-26)	24 (19-26)	U
**Plasma**				
Detectable RNA (n/N)		0/12	0/8	0/4
Ct Values (Median; range)		U	U	U
**Rectum**				
Detectable RNA (n/N)		2/7	1/5	1/2
Ct Values (Median;range)		27.5 (27-28)	28	27

U: Undetected.

Throat swabs were obtained after 4–18 d (median 10) of illness.

Sputum were obtained after 4–17 d (median 9.5) of illness.

Plasma samples were obtained after 7–10 d (median 9) of illness.

Rectal swabs were obtained after 4–16.5 d (median 9.5) of illness.
